# Assessment of the nursing care product (APROCENF): a reliability and construct validity study**^1^**

**DOI:** 10.1590/1518-8345.1495.2860

**Published:** 2017-04-06

**Authors:** Danielle Fabiana Cucolo, Márcia Galan Perroca

**Affiliations:** 2 PhD, Professor, Departamento de Enfermagem, Universidade Federal de São Carlos, São Carlos, SP, Brazil.; 3 PhD, Adjunct Professor, Faculdade de Medicina de São José do Rio Preto, São José do Rio Preto, SP, Brazil.

**Keywords:** Validity of Tests, Nursing Assessment, Outcome and Process Assessment (Health Care), Validation Studies, Health Management, Hospital Administration

## Abstract

**Objectives:**

to verify the reliability and construct validity estimates of the "Assessment of nursing care product" scale (APROCENF) and its applicability.

**Methods:**

this validation study included a sample of 40 (inter-rater reliability) and 172 (construct validity) assessments performed by nurses at the end of the work shift at nine inpatient services of a teaching hospital in the Brazilian Southeast. The data were collected between February and September/2014 with interruptions. Cronbach's alpha and Spearman's correlation coefficients were calculated, as well as the intraclass correlation and the weighted kappa index (inter-rater reliability). Exploratory factor analysis was used with principal component extraction and varimax rotation (construct validity).

**Results:**

the internal consistency revealed an alpha coefficient of 0.85, item-item correlation ranging between 0.13 and 0.61 and item-total correlation between 0.43 and 0.69. Inter-rater equivalence was obtained and all items evidenced significant factor loadings.

**Conclusion:**

this research evidenced the reliability and construct validity of the scale to assess the nursing care product. Its application in nursing practice permits identifying improvements needed in the production process, contributing to management and care decisions.

## Introduction

Nursing work takes place in production subsystems, adapting and interacting constantly with other subsystems^1^.This dynamics produces emergent behaviors, besides unexpected situations related to health practice, which demand self-organization and (re)prioritization of the activities, which are characteristics of a complex adaptive system (CAS)^2^. This demands flexibility from nursing to respond to the needs required^3^.

Its organization is not linear, but influenced by factors related to individual needs, health technologies and care production^1^.The product that results - care - is intangible, and delivered and consumed at the moment the care is provided^4^.

The authors consider the construct investigated in this study, the nursing care product, to be the interaction between structural factors (human capital and support services) and work organization methods (care planning, patient/family care and multidisciplinary interaction) that intervene in the care process. The more favorably aligned these factors and methods are, the better the product will be assessed.

As a result of the productive transformation, the professionals need instruments to measure the means needed for nursing's main activity from the perspective of nursing care^2^, contributing to management and care decisions. Despite the development and increasing use of specific instruments to measure results of interest in the nursing area, in the literature, no approaches and/or scales were evidenced that permit assessing the product generated at the end of the shift, considering the interaction between the work structure and organization methods. Thus, the "Assessment of the nursing care product" scale (APROCENF in Portuguese) was developed to evidence the critical factors of care management and classify the impact in the activities delivered at the end of a work shift (product) and which involves attending to the needs of the patients/relatives and professionals.

Measuring scales support professional activities and are available to measure a range of phenomena. Nevertheless, many of these do not properly describe their psychometric properties^5^.The reliability and validity measures determine the accuracy and quality of the instruments, helping the nurses in their evidence-based practice^6^.

The reliability is related to the precision of a scale, that is, if its measures reflect the true values of the attributes accurately^7^.One of the aspects of the reliability, the internal consistency, assesses whether the subparts of a scale measure the same characteristic or attribute. Another, the inter-rater reliability, verifies the agreement among two or more independent raters on the score obtained^8^.

The validity indicates the extent to which the scale measures what it intends to measure. One of its modalities, the construct validity, tests hypotheses, demonstrating trust or not with regard to what was proposed in the theoretical perspective^8^.Thus, an imprecise, that is, an unreliable measure cannot measure the attribute in a valid manner. On the other hand, the reliability does not guarantee the validity^8^.

Is the APROCENF valid and reliable? How do the nurses perceive its applicability in the care and management context? To answer these questions, this research was developed to verify reliability estimates and the construct validity of the "Assessment of the nursing care product" scale (APROCENF) and its applicability.

## Methods

The APROCENF scale rests on the concept of care production^1^, management^9^ ^)^ and quality in health^10^, combining factors that can contribute to the provision of nursing care. It consists of eight items: Nursing care planning; Resources needed for care provision; Estimation of nursing staff requirements; Educative actions and staff development; Care monitoring and transfer; Interaction and multidisciplinary activity; Patient and/or family care and Meeting the care needs. Each item is scored between one and four in which, the higher the score, the better the product of nursing care.

To apply the scale, the nurse needs to assess all items at the end of the work shift at one of the four levels, considering the option that best approaches the reality experienced. The individual item scores are added up and lead to a classification according to the following intervals: 9-12points (poor), 13-21(regular), 22-30 (good) and 31-36 (excellent). The content validity of the scale and its items was confirmed in advance^11^.

To verify the inter-rater reliability estimate and the construct validity, 40 and 172 nursing assessments at the end of the work shift were considered, respectively. The sample size for the validity assessment complied with the criterion estimated for the factorial analysis, i.e. 20 times the number of instrument items^12^.

The research was developed at three medical and surgical clinical inpatient services and six specialized services, being one Pediatric, one Maternal-Infant and four Intensive Care Units -ICU (General, Coronary, Pediatric and Neonatal) at a large teaching hospital in the Brazilian Southeast. The data collection for this phase of the study took place between February and September/2014.

The instrument was applied by clinical nurses, who were personally invited by one of the researchers and received individual advice on the objective of the instrument, its content and how to apply it.

Two pairs of nurses from two ICUs independently assessed 20 shifts each (inter-rater agreement)^8^. To assess the construct validity, 13 nurses applied the scale at the end of their work shifts. Each assessment was registered daily on a print scale the nurses received.

To investigate the nurses' perception on the use of the scale in professional practice, a questionnaire was applied with demographic and professional data and the participants also answered nine propositions on a five-point Likert scale (from I completely disagree to I completely agree), related to the pertinence, clarity, objectivity, complexity, grade, applicability and relevance of the instrument.

Descriptive statistics were used to characterize the sample and examine the frequency distributions for each item. The scale was considered as an ordinal measuring level and significance was set at p≤0.05for all analyses made.

To assess the internal consistency, the following were analyzed: Cronbach's alpha coefficient >0.70^8^,Spearman's correlation coefficient (item-item and item-total) with0.30 cut-off^13^and intraclass correlation coefficient (ICC) ≥0.70^8^.The inter-rater agreement level on the different classifications (bad, regular, good and excellent) was verified, considering the weighted Kappa coefficient(Kw), as follows:<0.20 (poor), 0.21-0.40 (fair), 0.41-0.60 (moderate), 0.61-0.80 (good) and 0.81-1.00 (very good)^14^.

The exploratory factorial analysis (EFA) using principal component extraction and the Varimax rotation method was applied for the construct validation. Initially, the Kaiser-Meyer-Olkin (KMO) index was calculated for each item and the overall scale and Bartlett's sphericity test was applied to verify the null hypothesis that there is no correlation among the items;

To define the number of factors extracted, the following were examined: eigenvalues superior to 1.0; *Scree plot* and whether the components represented at least 60% of the explained variance. Factor loadings ≥0.40 were considered and were only repeated if they loaded significantly in one factor (at least 0.20 of difference among the loadings). The communalities were assessed for each item and considered significant when>0.50^12^. Kruskal-Wallis' test was applied, followed by Dunn's post-test for multiple comparisons between the work shifts and the mean instrument score. For these analyses, the software Statistical Package for Social Sciences (SPSS, Chicago, Illinois*,* USA) 19.0 was used.

Approval for the research was obtained from the Research Ethics Committees of the institutions involved-process No. 0379/11 (Hospital1), process No.0050/12 (inclusion Hospital 2 and 3) and process No.002/12 (Hospital 3) and consent was obtained from the participating institutions and nurses.

## Results

### The assessments

The EFA was conducted using 172 assessments at medical and surgical clinical inpatient units (94;54%), Pediatric (26;14%) and Maternal-Infant units (13;8%),General ICU (13;8%), Coronary ICU (13;8%)and Pediatric ICU (13;8%), distributed among the following shifts: morning (39;23%), afternoon (76;44%) and evening (57;33%).Two assessments were excluded because they were incomplete. On average, each nurse performed 13 assessments. Most of the nurses were female (n=11), with an average age of 33 years (sd=5.2; range 28-43 years) and mean length of professional experience 5.5 years (sd=3.4; range 3-16 years).

The nursing care product was classified as poor (3; 1.8%), regular (38; 22.1%), good (111; 64.5%) and excellent (20; 11.6%), with a median of 24 points (Q1-Q3=21-26), equivalent to a good score. The item with the worst assessment (adding up poor and regular scores) was Interaction and multidisciplinary activity (103; 59.9%) and the best score (adding up good and excellent scores) went to Meeting the care needs(145; 84.4%).

The answers to the questionnaire on the pertinence, clarity, objectivity, complexity, grade, applicability and relevance of the instrument revealed lesser agreement on the propositions related to the common language among the professionals (Md4.0;Q1-Q3=4.0-5.0),clarity in the statements (Md 4.0; Q1-Q3=4.0-5.0) and implementation in daily practice (Md 4.0; Q1-Q3=4.0-4.0), but did not signal justifications and/or suggestions for improvements (Table 1).

**Table 1:** Opinion of the nurses on the Assessment of the Nursing Care Product(APROCENF) scale (n=13).Campinas, SP, Brazil, 2015

Proposition	Md (Q1-Q3)*
Discusses the most important items	5.0(4.0-5.0)
Discusses relevant factors of each item	5.0(4.0-5.0)
Permits a shared language among the professionals	4.0(4.0-5.0)
Presents clear statements	4.0(4.0-5.0)
Is very extensive	2.0(2.0-4.0)
Is complex	2.0(2.0-4.0)
The intensity of its grades increases	5.0(4.0-5.0)
Can be introduced in daily nursing practice	4.0(4.0-4.0)
Can produce useful data for management decision making	5.0(4.0-5.0)

*Md-median.Q1-Q3-quartiles

In the scores obtained per work shift, the afternoon period presented the highest median score (Md 25.0; Q1-Q3=22.5-28.0), followed by the morning shift (Md 23.0; Q1-Q3=19.0-24.0) and night shift (Md 22.0; Q1-Q3=20.0-26.0), with p<0.0001. The difference between the scores was confirmed using Dunn's post-test, which intends to explain the inter-group differences. Significance was found between the afternoon shift and the other, and no significance between the morning and night shifts.

### Reliability Test

The instrument presented a high Cronbach's alpha coefficient, equaling 0.85. The item-item correlation ranged between0.13 and 0.61.Correlations inferior to 0.30 involved the items: resources needed for care provision and interaction and multidisciplinary activity. The item-total correlation ranged between 0.43 (interaction and multidisciplinary activity) and 0.69 (patient and/or family care) (Table2).

**Table 2:** Spearman item-item and item-total correlation coefficient of the Assessment of Nursing Care Product (APROCENF) scale (n=172). Campinas, SP, Brazil,2015

Items*	Item1	Item2	Item3	Item4	Item5	Item6	Item7	Item8	Item-Total
Item1									0.67^†^
Item2	0.39^†^								0.46^†^
Item3	0.45^†^	0.34^†^							0.51^†^
Item4	0.47^†^	0.13^§^	0.42^†^						0.60^†^
Item5	0.52^†^	0.30^†^	0.38^†^	0.47^†^					0.68^†^
Item6	0.34^†^	0.20^‡^	0.25^‡^	0.31^†^	0.36^†^				0.43^†^
Item7	0.52^†^	0.41^†^	0.45^†^	0.45^†^	0.58^†^	0.33^†^			0.69^†^
Item8	0.45^†^	0.42^†^	0.42^†^	0.61^†^	0.59^†^	0.22^‡^	0.49^†^		0.68^†^

*Item 1-Nursing care planning; Item 2-Resources needed for care provision; Item 3-Estimation of nursing staff requirements; Item 4-Educative actions and staff development; Item 5-Care monitoring and transfer; Item 6-Interaction and multidisciplinary activity; Item 7-Patient and/or family care and Item 8-Meeting the care needs.

†p<0.0001;‡p<0.05;§p<0.1

### Inter-rater reliability

Forty assessments were considered, undertaken by two pairs of nurses working at the Neonatal and Coronary ICU, to confirm the agreement. The inter-rater equivalence, represented by the ICC, ranged between 0.71 (95% CI: 0.36-0.88) and 0.93(95% CI: 0.83-0.97) and the Kw between 0.23 (95% CI: -0.06-0.52) and 0.83 (95% CI: 0.61-1.00). Eight disagreements were observed in one pair of nurses, but none of them was superior to one degree (one classification immediately superior or inferior) in relation to the total score.

### Construct validity

The KMO measure of sample adequacy was equal to 0.85, ranging between 0.76 and 0.92, and Bartlett's sphericity test was significant at p<0.01.Then, the adjustment of the data to the factorial analysis was verified. The correlation matrix indicated different items with coefficients≥0.30.

An EFA model with eight factors was adjusted and only the first factor presented an eigenvalue>1,confirmed in the Scree plot. Nevertheless, two factors explained60.2%of the accumulated variance. Factor 1 (eigenvalue 3.98) explained 49.7% of the total variance and factor 2 (eigenvalue 0.84) 10.5%. The two-factor model was then adjusted and the factor loadings were analyzed, demonstrating no precise association of all items with the factors (at least 0.20 of difference among the loadings). Therefore, only the first factor was considered for interpretation (Table 3).

**Table 3:** Varimax rotated factorial matrix considering single and two-factor models (n=172). Campinas, SP, Brazil, 2015.

Items*	Two-factor model			Single-factor model
	Factor1	Factor2		Factor1
Item1	0,60	0,49		0,77
Item2	^†^	0,90		0,57
Item3	0,46	0,42		0,62
Item4	0,87	^†^		0,71
Item5	0,74	0,32		0,79
Item6	0,31	0,48		0,53
Item7	0,62	0,49		0,79
Item8	0,74	0,32		0,79

*Item 1-Nursing care planning; Item 2-Resources needed for care provision; Item 3-Estimation of nursing staff requirements; Item 4-Educative actions and staff development; Item 5-Care monitoring and transfer; Item 6-Interaction and multidisciplinary activity; Item 7-Patient and/or family care and Item 8-Meeting the care needs.

†Factor loading inferior to 0.30

## Discussion

To produce good outcomes in health care, the organization and delivery of the care needs to be improved, in order to comply with the safety, equity, punctuality, efficiency and efficacy dimensions with a focus on the needs of the subjects involved^15^. The nurses are responsible for the management dimensions of care and should carefully assess this process, proposing improvements for practice^16^.

The availability of valid and reliable instruments enhances the accuracy of the information collected, as well as the efficacy of the decision process^17^ ^-^ ^18^. Therefore, assessing the psychometric properties, including the reliability and construct validity of the APROCENF scale, has become essential.

In the reliability analysis, the correlation coefficient determines the intensity of the relation between the instrument items (item-item) and between each item and the total score (item-total), using 0.30 as the cut-off for validation^13^.Although this criterion was not fully complied with, correlations≥0.30were found with other items, justifying its maintenance in the instrument. The assessment of the Cronbach's alpha coefficient when deleting the item for item 2, related to the Resources needed for care provision, and for item 6 (Interaction and multidisciplinary activity), evidenced no significant interference in the internal consistency of the instrument. In addition, these are important variables for care production from the perspective of CAS^2^ and the patient safety concept^19^.Thus, the instrument continued with eight items. As regards the ICC, in view of the score, equivalence was obtained.

The nursing care product at the end of the work shift is the abstract concept under analysis and the construct validity investigated the adequacy of the instrument to measure it. The factor loadings represent the item's correlation with the factors, with significance when ranging between 0.50 and 0.70 and indicating a well-defined structure when>0.70^12^.

The findings revealed significant factor loadings in all scale items. The dimensions of patient and family-centered care (Meeting the care needs and Patient and/or family care) seem to strongly influence the assessment of the nursing care product. This health care strategy acknowledges the importance of the relations and promotes the partnership between professionals and patients, encouraging autonomy and the patients' engagement in the decisions and in care planning. In addition, the strategy advocates better communication skills, through the development of reflexive listening and an environment that permits the teams' engagement^20^.

Some items related to the structure (Estimation of nursing staff requirements and Resources needed for care provision) and relationships (Interaction and multidisciplinary activity) presented a lower weight in the factor analysis and communalities of 0.38, 0.33 and 0.28, respectively. These items were not determinant, but are significant in the composition of the scale. It can be inferred that the factorial structure does not explain much of the variation in these items.

Considering that, the more adjusted the structural factors and the work organization methods, the better the nursing product will be. In the nurses' assessments at the end of the work shift, 64.5% evidenced a good care product. When comparing the scores per work shift, the afternoon period seems to indicate the best nursing care product. Nevertheless, new studies are needed to explain the shift work and the care provision.

The items with favorable weights, adding up good and excellent, were Meeting the care needs (84.4%) and Estimation of nursing staff requirements (76.7%). As for Meeting the care needs, the accomplishment of planned interventions and nurses' specific activities with minimization of patient risks were considered. The health quality programs have been widespread in the past decade, mainly in hospitals. Studies have demonstrated a significant association between the quality of care and/or unattended needs and the occurrence of adverse events^10^and the nurses working at these institutions prioritize patient care management, mitigating these risks^21^,also evidenced in this study. In the same sense, the adequate composition of the nursing team to respond to the patients' demands has been widely discussed and studied, due to its close relation with the quality and safety of care^22^.Nevertheless, it is important to highlight that this is a picture of the practical scenario under investigation and that new studies in other contexts can diverge.

On the other hand, the items with negative weights, adding up regular and poor, are related to Interaction and multidisciplinary activity (59.9%) and Care monitoring and transfer (28.5%). These questions seem to advance timidly in view of the complexity of the interventions and the need for knowledge articulation and team integration^23^.Therefore, teaching and interdisciplinary practice have been investigated, and are currently encouraged to improve the fragmented health systems, constituting a feasible and efficient service provision model^24^.Another challenge evidenced in this study that is in accordance with other findings^25^ is related to the transfer of information among clinical teams through formal registers, reducing adverse incidents and improving the continuity of health care.

It can be highlighted that the scale permits not only measuring the nursing care product at the end of each work shift, but also mapping the critical aspects that compromise the productive system. Thus, it equips nurses, health managers, educators and researchers by targeting proposals for improvement towards the weaknesses evidenced.

According to the nurses' perception on the use of the scale in professional practice, the instrument is pertinent, relevant and supports management decision taking. They agree on the clarity and applicability as well, but argue some difficulty in using it daily, in view of the activities related to the work process, supporting earlier assessments^11^.

As all production systems aim for continuous improvement, this research was intended to contribute to the discussions on the productive transformation in nursing, targeting the management of patient-centered care. Although evidence of reliability and construct validity were found when the scale was used at different health care units, additional studies are needed to examine the feasibility of its application in other contexts. Another limitation in this research was the non-random selection of the participating nurses and work shifts. The Kappa coefficients found (Kw equal to 0.23-95%CI: -0.06-0.52) in one pair of raters were considered poor/fair.

## Conclusion

In this research, the reliability and construct validity of a tool to assess the nursing care product were evidenced. Its application in clinical practice allows the nurse managers to measure the efficiency and efficacy of the production process, contributing to management and care decisions.
